# Proteomics analysis of serum protein patterns in duck during aflatoxin B1 exposure

**DOI:** 10.14202/vetworld.2019.1499-1505

**Published:** 2019-09

**Authors:** Natthasit Tansakul, Jatuporn Rattanasrisomporn, Sittiruk Roytrakul

**Affiliations:** 1 Department of Veterinary Pharmacology and Toxicology, Faculty of Veterinary Medicine, Kasetsart University, Bangkok, Thailand; 2 Department of Companion Animal Clinical Sciences, Faculty of Veterinary Medicine, Kasetsart University, Bangkok, Thailand; 3 Proteomics Research Laboratory, National Center for Genetic Engineering and Biotechnology, 113 Thailand Science Park, Klongluang, Pathumthani 12120, Thailand

**Keywords:** aflatoxin B1, duck, proteomics, serum

## Abstract

**Background and Aim:**

Unlike the already well-documented human serum proteome, there are still limitations regarding analyzing and interpreting the various physiological changes and disease states of the serum proteomes found in duck. Serum proteome in duck under the condition of mycotoxin contamination in feed has not yet been examined. This study aimed to introduce the characterization of the circulating proteomes in duck serum during exposure to aflatoxin B1 (AFB1).

**Materials and Methods:**

Duck serum samples were collected from four experimental groups, gel-based mass spectrometry was then applied, and finally, 445 proteins were identified in pulled serum sample.

**Results:**

Among these 445 proteins, 377 were present in at least one group from all. There were 35 proteins which were expressed when the duck was exposed to AFB1. The protein library that allows the identification of a large number of different proteins in duck serum will be enhanced by the addition of these peptide spectral data. It is noteworthy that chromodomain-helicase-DNA-binding protein 7 (CHD7) [*Gallus gallus*] was up-regulated in the group with the highest AFB1 contamination.

**Conclusion:**

CHD7 protein might be somehow relative to aflatoxicosis in the duck that causes poor performance and economic loss. Moreover, other proteins present in duck serum were also added in the protein library.

## Introduction

Mycotoxin is commonly found in poultry feed. Incidence of mycotoxicosis could potentially cause several diseases associated with poor performance, impair immune or organ function and results in severe economic loss in the poultry industry. Poultry feeds in Thailand that is located in tropical zone are commonly contaminated with low level of aflatoxin. Aflatoxin B1 (AFB1) is known as a hepatotoxic and carcinogenic agent in human and several animal species.

Other reports have mentioned the ways in which poultry performance and serum profiles are affected by aflatoxin [1]. Among poultry, duck is the most aflatoxicosis susceptible species followed by turkey, broiler, and laying hen. Aflatoxicosis is commonly responsible for serum alteration and immunotoxicity in both terms of acute and chronic toxicity. Aflatoxin could also suppress immune system function even when exposure remains at low levels. Thus, avian flock may be easily infected by this pathogenic microorganism and shows poor performance associated with economic loss. The decreased total plasma proteins and albumin levels were the principal indices of aflatoxicosis in laying hens, as well as the increased prothrombin content [2]. Therefore, identification of specific serum protein profiles that are altered due to the aflatoxin consumption may lead to quick diagnosis, treatment, and prevention strategies for aflatoxicosis in poultry.

At present, works that explore avian serum proteome characterization are scarce. So far, serum proteomics evaluations of chicken for *Eimeria* spp. or avian pathogenic *Escherichia coli* exposure and hen physiology during growth were reported [3,4]. In duck, proteomics analysis has been already performed after viral infection [5]; the proteomes in liver of Pekin duck [6] and duck meat [7] are also examined. In a previous study, research was conducted on the proteomics and metabolomics analysis of proteins related to reproductive performance in Muscovy duck [8]. However, information on the characterization of serum proteome in duck has not yet been investigated with regard to the condition of feed contaminated by mycotoxins. An exploration of the composition of proteins found in serum can facilitate the identification of protein biomarkers that can add specific economic value to animal production and insights into the interactions involving aflatoxin and the molecular mechanisms involved in avian pathogenesis.

This study aimed to introduce the characterization of the circulating proteomes in duck serum during exposure to AFB1.

## Materials and Methods

### Ethical approval

The “Ethical Principles for the Use of Animals for Scientific Purposes” that were published by the National Research Council of Thailand and received the approval of the Kasetsart University Animal Research Committee were applied to the study (TRF-MG56).

### Preparation of the animals for the sample

Forty-eight 6-week-old ducks were the animals used in the present study and were kept under identical standard conditions and provided with feed and water *ad libitum*. Ducks were exposed to natural daylight and 5 lux LED light during night. The room temperature was set between 30°C and 32°C with relative humidity at 60-65% through experiment. After 1 week of acclimatization, ducks were equally allocated to four experimental groups. As known, natural feeds are not absolutely free from mycotoxin contamination. Therefore, five major mycotoxins including AFB1, deoxynivalenol, zearalenone, ochratoxin A, and T-2 toxin were determined by reverse-phase high-performance liquid chromatography tandem-mass spectrometry (LC-MS/MS) following the in-house validated method. Feed that contained AFB1 at a level <25 µg/kg feed and did not include harmful level of other mycotoxins was used as the control feed in the experiment. Group 1served as the control group and was fed with this diet. Group 2 was fed with the same diet and was also orally administered with AFB1 once per day at the dose of 100 µg/kg body weight (BW). Group 3 and Group 4 were provided with aflatoxin-contaminated feed at the levels of 100 and 250 µg/kg feed for 10 days, respectively. Serum was collected at days 5 and 10 post-treatment and stored into tubes containing ethylenediaminetetraacetic acid, centrifuged and then kept in −80°C until analysis.

### Gel-based MS approaches

#### Pre-fractionation protein by sodium dodecyl sulfate-polyacrylamide gel electrophoresis (SDS-PAGE)

The fractionation of proteins was conducted on SDS-PAGE mini slab gel (8 cm×9 cm×0.1 cm, HoeferminiVE, Amersham Biosciences, UK). Preparation of this polyacrylamide gel was carried out in accordance with the standard method according to Laemmli [9]. Lowry’s method was employed to determine the total serum protein concentration. At each time point, 20 µg of pooled serum protein were obtained from six ducks per group, and then the fractionation on the gel was performed. The separating gel used for the fractionation contained 12.5% acrylamide. After the protein samples were divided into equal amounts and combined with 5 µl of 5× sample buffer (0.125 M Tris-HCl pH 6.8, 20% glycerol, 5% SDS, 0.2 M DTT, and 0.02% bromophenol blue), they were boiled at 95°C for 10 min, and they were then placed onto the 12.5% SDS-PAGE. The size of the polypeptides was estimated through the use of the low molecular weight protein standard marker (Amersham Biosciences, UK). The performance of the electrophoresis in the SDS electrophoresis buffer (25 mM Tris-HCl pH 8.3, 192 mM glycine, 0.1% SDS) run until the tracking dye reached bottom of the gel. Visualization of the protein bands was carried out using staining with silver in accordance with Blum [10]. After staining, a gel was sliced into 12 pieces following molecular band. After reduction and alkylation, the in-gel digestion with the in-house method [11] was then applied to the gel pieces before the analysis of LC-MS/MS was conducted. Then, the Mascot program was used to submit this data to the database search.

#### In-gel digestion

Following the excising of the protein bands, dehydration of the gel plugs with 100% acetonitrile (ACN) and reduction with 10mM DTT in 10 mM ammonium bicarbonate at room temperature for 1 h and alkylation at room temperature for 1 h in the dark in the presence of 100 mM iodoacetamide in 10 mM ammonium bicarbonate were carried out. Following the alkylation, 100% ACN was used to dehydrate the gel pieces for 5 min twice. The in-gel digestion of proteins was achieved by the addition of 10 µl of trypsin solution (10 ng/µl trypsin in 50% ACN/10 mM ammonium bicarbonate) to the gels, after which they were incubated for 20 min at room temperature, and then, to maintain the immersions of the gels throughout the digestion, 20 µl of 30% ACN was added. The incubation of the gels was continued for several hours overnight at 37°C. Extraction of the products of the peptide digestion was conducted by the addition of 30 µl of 50% ACN in 0.1% formic acid (FA) to the gels, and incubation of the gels was undertaken in a shaker at room temperature for 10 min. The extracted peptides were collected, and a new tube was used to pool them together. A vacuum centrifuge was used to dry this pool of extracted peptides, which was then stored at −80°C to conduct additional mass spectrometric analysis at a later time.

### HCT-ultra LC-MS analysis

Analysis of the peptide solutions was performed using the HCT-Ultra PTM Discovery System (Bruker Daltonics Ltd., U.K.) in combination with an UltiMate 3000 LC System (Dionex Ltd., UK). Separation of the peptides was done on a nanocolumn (PepSwift monolithic column 100 μm i.d.× 50 mm). For eluent A, 0.1% FA was used, and 80% ACN in water containing 0.1% FA was used as eluent B. Achievement of peptide separation was reached using a linear gradient from 10% to 70% B for 13 min at a flow rate of 300 nl/min that at 90% B included a regeneration step and at 10% B, an equilibration step with one run lasting 20 min. Acquisition of the peptide fragment mass spectra took place in the data-dependent AutoMS mode with a scan range of 300-1500 m/z, three averages, and as many as five precursor ions chosen from the MS scan of 50-3000 m/z. Detection and deconvolution of the peptide peaks were automatically conducted with the DataAnalysis version 4.0 (Bruker Daltonics, Bremen, Germany). The creation of mass lists in the Mascot Generic File format was also automatically done, and these lists were input into the searches of Mascot MS/MS Ions in the non-redundant database of the National Center for Biotechnology Information (NCBI nr) (www.matrixscience.com). The following default search parameters were used: Enzyme = trypsin, max. missed cleavages =1; fixed modifications = carbamidomethyl (C); variable modifications = oxidation (M); peptide tolerance ±1.2 Da; MS/MS tolerance ±0.6 Da; peptide charge = 1+, 2+, and 3+; and instrument = ESI-TRAP.

The quantitation of the proteins employed DeCyder MS Differential Analysis software (DeCyderMS, GE Healthcare). Conversion of the acquired LC-MS raw data was undertaken and automated peptide detection, charge state assignments, and quantitation based on the intensity of the peptide ions signals in MS mode were performed using the PepDetect module. The Mascot software (Matrix Science, London, UK) was used for submission of the MS/MS data that were analyzed by the DeCyderMS to carry out the search of the database. The presence or absence of each identified protein was determined by each group’s maximum value. Analysis of the protein expression ratio was performed through a comparison of the various group’s protein intensity values. The number of candidate proteins was reduced to search for the potential diagnostic markers for duck aflatoxicosis by identifying only the proteins that had a minimum of two peptides from MASCOT as true matches that could be considered as acceptable. The NCBI database was used to identify the proteins in the data. Taxonomy (Human or Eucaryote); enzyme (trypsin); variable modifications (carbamidomethyl and oxidation of methionine residues); mass values (monoisotopic); protein mass (unrestricted); peptide mass tolerance (1 Da); fragment mass tolerance (±0.4 Da), peptide charge state (1+, 2+, and 3+), and max missed cleavages were assigned for the interrogation of the database. To be considered as identified proteins, it was required that the proteins had the minimum two peptides with an individual mascot score of p<0.05 and p<0.1, respectively.

## Results

As a result of the Mascot search of human and avian data, 445 differentially expressed proteins were found. These differentially expressed proteins were revealed by hierarchical clustering analysis (Data not showed).

**Figure-1:**
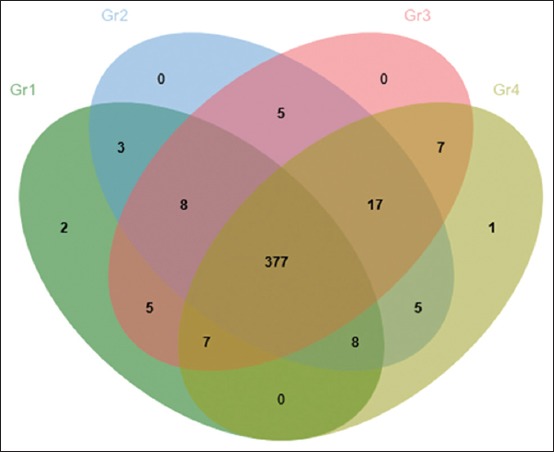
Venn diagram illustrates the number of differentials expressed proteins found in the experimental groups of ducks.

Duck serum samples were collected from four experimental groups, following gel-based MS approached, amount of 445 proteins were identified in pulled serum sample (Figure-1). Of those 445 proteins, 377 proteins were present in at least one from all groups. There were 35 proteins which were expressed when duck exposed to AFB1. At the high dose of AFB1 contamination (Gr3 and Gr4), there were 8 proteins expression. As illustrated in Figure-1, there was a single protein upregulated only in Gr4; chromodomain-helicase-DNA-binding protein 7 (CHD7) [*Gallus gallus*].

**Figure-2:**
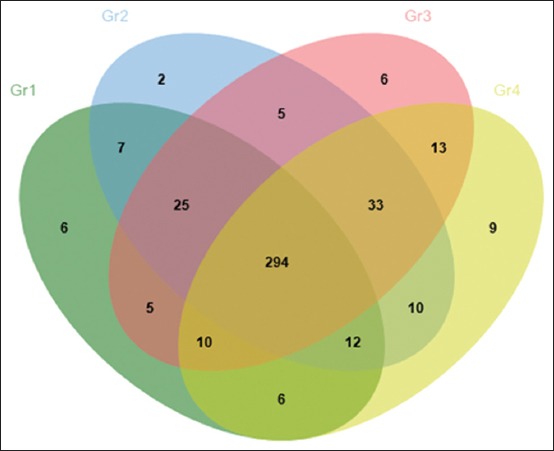
Venn diagram shows the number of differentials expressed proteins found between groups of duck after day 5 of aflatoxin B1 exposure.

**Figure-3:**
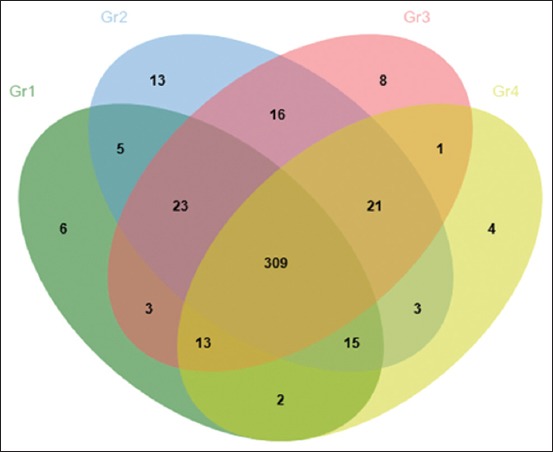
Venn diagram shows the number of differentials expressed proteins found between groups of duck after day 10 of aflatoxin B1 exposure.

Venn diagram shows the number of differentials expressed proteins found between groups of duck after day 5 (Figure-2) and day 10 (Figure-3) of aflatoxin exposure. As indicated in Figure-2, after 5 days AFB1 exposure, there were 9 proteins upregulated in Gr4, including coiled-coil domain-containing protein 57 [*Anasplatyrhynchos*], protein Wnt-9b [*G. gallus*], AT-rich interactive domain-containing protein 4A [*G. gallus*], stAR-related lipid transfer protein 8-like [*Meleagris gallopavo*], CHD7 [*G. gallus*], zinc finger matrin-type protein 1-like [*A. platyrhynchos*], OTU domain-containing protein 7B-like [*M. gallopavo*], BTB/POZ domain-containing protein 3, partial [*A. platyrhynchos*], and C-C motif chemokine 4-like [*M. gallopavo*].

As shown in Figure-3, following 10 day of AFB1 exposure, there were 4 proteins upregulated in Gr4, including 26S proteasome non-ATPase regulatory subunit 4-like isoform X2 [*G. gallus*], putative interleukin 17 receptor E-like, partial [*A. platyrhynchos*], CHD7 [*G. gallus*], and sodium bicarbonate cotransporter 3-like [*M. gallopavo*].

The preliminary results of five ontology classifications including molecular function, biological process, cellular components, protein class, and pathway class were classified. Functional protein classifications by molecular function were composed of catalytic activity (44%), binding (30%), receptor activity (17%), and structural molecule activity (9%). The categorization of biological function of protein expression provided 12 different component ratios which were a majority group of cellular process (30.60%), followed by metabolic process (26.50%), multicellular organismal process (10.20%), cellular component organization or biogenesis (8.20%), developmental process (6.10%), response to stimulus (6.10%), biological regulation (6.10), immune system process (4.10), and localization (2.00%). Categorization of the most abundant proteins by cellular components composed of membrane (20%), followed by organelle (27%), cell part (26%), macromolecular complex (13%), cell junction (7%), and extracellular region (7%), respectively.

In case of distribution of the proteins by protein class, a majority protein function was nucleic acid binding (17%) while other minor classes were receptor (13%), hydrolase (7%), cell junction protein (7%), enzyme modulator (7%), lyase (7%), cytoskeletal protein (7%), transferase (7%), transcription factor (7%), transfer/carrier protein (3%), ligase (3%), calcium-binding protein (3%), oxidoreductase (3%), isomerase (3%), transporter (3%), and signaling molecule (3%). There were five categories which equally abundant proteins shown under pathway class; Ras Pathway (20%), integrin signaling pathway (20%), CCKR signaling map (20%), TCA cycle (20%), and vasopressin synthesis (20%).

**Figure-4:**
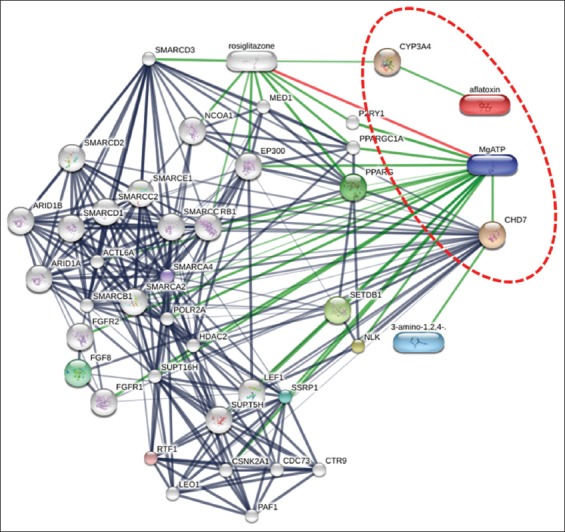
The Stitch4.0 software (http://stitch.embl.de/) was applied to predict the biological protein-protein interaction networks of the differential abundance proteins expressed on duck serum that was contaminated using aflatoxin B1. The thicker lines are used to present the associations that are stronger. The blue line represents the protein-protein interactions, the green line shows the chemical-protein interactions, and the red line indicates the interaction between chemicals.

The summary of the biological interaction of the proteins that were identified involved the construction of a protein-protein functional network with the use of the online software Stitch4.0 (http://stitch.embl.de/). Figure-4 shows the differentially expressed proteins on duck serum network. A more detailed view of the complex framework of proteins that could be a cause of the variations in aflatoxicosis in the duck was revealed by the analysis of this protein network.

## Discussion

Among poultry, duck is the most aflatoxicosis sensitive species followed by turkey, broiler, and laying hen. Other reports have described how poultry performance and serum profiles are affected by aflatoxin [1]. In addition, total serum protein and albumin may decrease after ingested feed with aflatoxin [2]. Aflatoxin could also suppress immune system function even when exposure remains at low levels. Thus, avian flock may be easily infected by pathogenic microorganism and shows poor performance associated with economic loss.

At present, proteomics is one of the advanced research fields offering a new perspective to an expression of protein and it is a field that falls under functional genomics and the context-defined analysis of complete complements of proteins [12]. Proteomic techniques for identification of certain protein patterns in poultry after challenged with endotoxin have recently been developed [13]. In the field of research concerning mycotoxin, proteomics is being increasingly applied [14].

In animal, little information on serum proteome characterization is available. Recently, a proteomics method using duck as a model to study protein expression of viral infection has been reported [5]. However, works that explore the proteome of duck serum have not been conducted to date. Differentially expressed proteins of healthy ducks and ducks exposed to aflatoxin performed by gel electrophoresis and tandem mass fingerprinting by LC-MS/MS may provide the insight to characterize proteome in the sera and lead to the understanding of molecular mechanisms of aflatoxicosis. The identification of the novel protein biomarkers with specific economic value in animal production may be accelerated by exploration of the composition of proteins in serum.

Identification of a total of 445 proteins was accomplished in the pulled serum samples in this study (Figure-3). From these 445 proteins, 377 proteins were found in one or more of all of the groups. There were 35 proteins which were expressed only when the duck was exposed to AFB1. In this study, there was no identification of apolipoproteins AII, haptoglobin or antitrypsin. Although it has been reported that haptoglobin was found in birds, including chickens, apolipoproteins AII has not been found to exist in avian serum. It was widely reported that changes of blood serum proteins in chickens during the fattening process in terms of their concentration and fractions occur, and it was concluded that changes of concentration of individual fractions did not always follow the quantity of total proteins in the blood serum [15].

As indicated, two proteins were downregulated after exposure to the medium and high dose of aflatoxin (Gr2 and Gr3); ADNP homeobox protein two isoform X1 [*A. platyrhynchos*] and mitochondrial ornithine transporter 1 [*G. gallus*]. These two proteins could serve as biomarkers of acute aflatoxicosis. It has been reported that, at an early stage of the pathological alteration in the neurodegeneration disease of humans, the downregulation of protein ADNP expression is observed [16]. The ADNP gene is upregulated in normal proliferative tissues. A family of eukaryotic intracellular transport proteins is constituted by the mitochondrial carriers (MCs) that function the transportation nucleotides, amino acids, carboxylic acids, inorganic ions, and cofactors that play a major role in oxidative phosphorylation, transfer of reducing equivalents of NADH, gluconeogenesis, and fatty acid metabolism in addition to mitochondrial replication, transcription, and translation, with few exceptions. Their physiological importance is underlined by the fact that mutations in several MCs are responsible for human diseases [17].

As illustrated in the Venn diagram (Figure-1), a single protein, the upregulated protein CHD7 [*G. gallus*], was found in the AFB1 contaminated group at the level of 250 µg/kg feed (Gr4). Interestingly, two proteins were downregulated in the group orally supplemented with AFB1 at the dose of 25 µg/kg BW (Gr1); ADNP homeobox protein 2 isoform X1 [*A. platyrhynchos*], and mitochondrial ornithine transporter 1 [*G. gallus*].

It is interesting to note that one protein, CHD7 [*G. gallus*], has been identified in this study and has been found upregulated in only Group 4 (the highest AFB1 concentration). A class of ATP-dependent chromatin remodeling enzyme that functions in the invoking changes in the interaction between DNA and nucleosomes is represented by the CHD. It is a factor in cellular processes, for example, replication, transcription, recombination, repair, and development [18].

The CHD7 is an ATP-dependent chromatin-remodeling link that regulates polymerase II transcription as well as the specific step of polymerase I transcription termination. In addition, it may regulate negatively DNA replication (UNIPORT). Mutation of the CHD7 gene has been reported to be linked to “CHARGE” syndrome in human embryo which “R” refers to retardation of growth and development when other abbreviation letters are ignorable [19].

One group of heme-thiolate monooxygenases is comprised of cytochromes P450. CYP3A4 (cytochrome P450, family 3, subfamily A, and polypeptide 4) is involved in aflatoxin metabolism as observed in the networks of the protein-protein interactions (Figure-4). In addition, this enzyme is linked to an NADPH-dependent electron transport pathway in liver microsomes. It is responsible for various oxidation reactions of structurally unrelated compounds, such as fatty acids, steroids, and xenobiotics. That CYP3A4 plays a major role in the biotransformation of AFB1 into the toxic product AFB1-8,9-epoxide. Moreover, AFB1 bioactivation in poultry species is caused by CYP2A6, CYP3A37, CYP1A5, and CYP1A1. Interindividual variations in the activation of aflatoxins rate in various species are also present.

Therefore, CHD7 might be somehow relative to aflatoxicosis in the duck that results in poor performance and economic loss. In this study, the low resolution of the 1-D analysis may cause the failure to detect these proteins. Thus, increased enhancement of the resolution of 2-D analysis will be necessary for future studies. Furthermore, because the proteome is dependent on context, defining the precise state of the animals that the plasma is extracted from is critical.

In the future, further independent validation of these biomarkers should be confirmed using greater number of samples and more techniques including Western blot to obtain the greatest diagnostic power for the assessment of aflatoxicosis in duck from the healthy duck.

## Conclusion

In summary, this is the first study to achieve the establishment of a database of serum proteins of duck following exposure to AFB1. The information that is necessary in case of the additional exploration of the levels and patterns of duck serum proteins using different forms of administration and with various concentrations of the toxin is provided by this database. Based on the changes in the protein levels that occur during development, it is suggested that there is the possibility that they have an effect on the stage of exposure to aflatoxins. At present, evaluation of these proteins as candidates for uses in future investigations of markers for aflatoxicosis in duck is being conducted.

## Authors’ Contributions

NT designed the experimental study and wrote the manuscript. JR supported to prepare pre-fractionation protein by SDS-PAGE and samples for MS analysis. SR was responsible for proteomics part and assisted for data analysis. All authors contributed to the submitted manuscript.
